# Exercise Ameliorates Atherosclerosis via Up-Regulating Serum β-Hydroxybutyrate Levels

**DOI:** 10.3390/ijms23073788

**Published:** 2022-03-30

**Authors:** Zhou Xu, Mingyue Zhang, Xinran Li, Yong Wang, Ronghui Du

**Affiliations:** 1 State Key Laboratory of Pharmaceutical Biotechnology, Jiangsu Key Laboratory of Molecular Medicine, Medical School, Nanjing University, Nanjing 210093, China; mg1935017@smail.nju.edu.cn (Z.X.); mg21350051@smail.nju.edu.cn (M.Z.); mg20350011@smail.nju.edu.cn (X.L.); 2 State Key Laboratory of Analytical Chemistry for Life Science, Nanjing University, Nanjing 210093, China

**Keywords:** atherosclerosis, exercise, BHB, ABCA1, ABCG1, SR-BI, autophagy, foam cell formation

## Abstract

Atherosclerosis, accompanied by inflammation and metabolic disorders, is the primary cause of clinical cardiovascular death. In recent years, unhealthy lifestyles (e.g., sedentary lifestyles) have contributed to a worldwide epidemic of atherosclerosis. Exercise is a known treatment of atherosclerosis, but the precise mechanisms are still unknown. Here, we show that 12 weeks of regular exercise training on a treadmill significantly decreased lipid accumulation and foam cell formation in ApoE^−/−^ mice fed with a Western diet, which plays a critical role in the process of atherosclerosis. This was associated with an increase in β-hydroxybutyric acid (BHB) levels in the serum. We provide evidence that BHB treatment in vivo or in vitro increases the protein levels of cholesterol transporters, including ABCA1, ABCG1, and SR-BI, and is capable of reducing lipid accumulation. It also ameliorated autophagy in macrophages and atherosclerosis plaques, which play an important role in the step of cholesterol efflux. Altogether, an increase in serum BHB levels after regular exercise is an important mechanism of exercise inhibiting the development of atherosclerosis. This provides a novel treatment for atherosclerotic patients who are unable to undertake regular exercise for whatever reason. They will gain a benefit from receiving additional BHB.

## 1. Introduction

Cardiovascular diseases caused by atherosclerosis, a chronic disease that correlates with inflammation and metabolic disorders, are still the leading cause of clinical cardiovascular death despite innovations in primary and secondary preventive measures [1,2]. Unhealthy lifestyles including, but not limited to, smoking, obesity, and a lack of exercise contribute to the worldwide epidemic of atherosclerosis [2,3].

Mechanistically, the accumulation of cholesterol and foam cell formation plays a critical role in the progress of atherosclerotic lesions [4,5]. During atherogenesis, scavenger receptors on the surface of macrophages, such as CD36 and scavenger receptor A (SRA), uncontrollably take up massive amounts of cholesterol. However, the scavenger receptor class B Type I (SR-BI), and the ATP-binding cassette transporters A1 and G1 (ABCA1 and ABCG1), which are key cellular cholesterol transporters of reverse cholesterol transport (RCT), cannot remove excess cholesterol out of cells, resulting in the accumulation of cholesterol esters and foam cell formation [4,6]. Enhancing the expression of ABCA1 and other cholesterol transporters increases macrophage cholesterol efflux, which is antiatherogenic [7,8].

Recently, many people have reported that cholesterol efflux, mediated by ABCA1, is dependent on autophagy [9]. Autophagy is an unselected self-digesting cellular pathway involved in organelle and protein degradation [10]. The cholesteryl ester (CE) accumulated in the cell must be transported in a free form, which is also the limiting step of RCT [11]. Autophagy can regulate cholesterol efflux by degrading CE to free cholesterol [9]. Promoting autophagy has become a novel pathway to promote lipid clearance [12].

Moderate exercise can reduce the risk factors of cardiovascular diseases and inhibit the development of atherosclerosis [3,13]. Exercise training improves the function of endothelial cells and promotes metabolism [14,15,16,17]. In earlier years, it was reported that exercise could increase plasma HDL levels, which is conceptualized as a biomarker of the antiatherogenic RCT process [18,19]. However, the precise mechanisms remain unknown.

After fasting and long periods of exercise, β-hydroxybutyric acid (BHB), the most abundant ketone body in mammals’ circulation, rises significantly [20,21]. The ketone body, mainly produced in the liver, serves as a necessary alternative energy source when the body encounters an energy crisis. In humans, BHB levels in the body are always at extremely low levels, at approximately 100–250 µM, and it can rise to 1–2 mM after fasting or prolonged exercise [22,23]. BHB suppresses inflammatory diseases by blocking NLRP3 inflammasome activation [24] and it can also suppress lipid accumulation via GPR109A-mediated signaling [25]. In addition, it has also been found to regulate the upstream protein of autophagy expression [26,27]. Exogenous BHB administration, such as via a ketogenic diet, has been used as a therapy in Alzheimer’s disease and obesity [28,29]. The effects of BHB on atherosclerosis are contentious and the exact mechanisms are still unknown, which all deserve further study.

In this study, we found that long-term regular exercise can significantly increase the levels of BHB in the serum of ApoE^−/−^ mice, fed with a Western diet, and that BHB treatment can upregulate the expression of lipid transporters, promote autophagy, and inhibit foam cell formation in vivo or in vitro. These data indicated that exercise may alleviate atherosclerosis by upregulating BHB levels in the serum.

## 2. Results

### 2.1. Regular Exercise Training Can Reduce Atherosclerotic Lesions and Increase Serum BHB Levels

**Figure 1:**
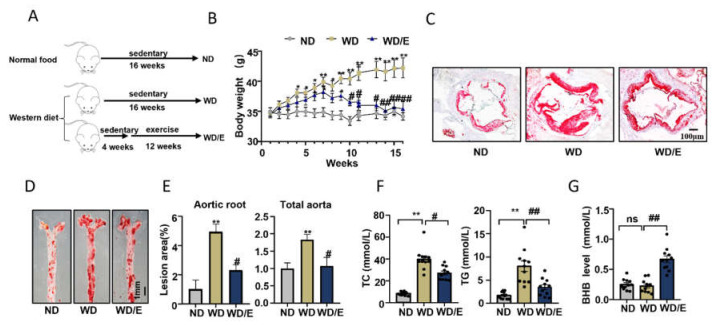
Exercise inhibits the development of atherosclerosis and increases serum BHB levels. (**A**) ApoE^−/−^ mice were fed normal food (ND) or a Western diet (WD), and either had access to exercise (E) for 12 weeks or did not (sedentary) (**B**) Body weight of mice (n = 11). (**C**) Representative images of the cross-sections of the aortic root stained with Oil Red O. Scale bars, 100 μm. (**D**) Representative images of the whole aorta stained by Oil Red O. Scale bars, 1 mm. (**E**) Quantification of cross-sections of the lesion area in the aortic root and whole aorta (n = 6). (**F**) Total cholesterol (TC) and triglycerides (TG) in the serum of ApoE^−/−^ mice (n = 11). (**G**) BHB levels in the serum from the mice (n = 11). Data are represented as means ± SEM. * *p* < 0.05, ** *p* < 0.01 vs. ND mice, and # *p* < 0.05, ## *p* < 0.01 vs. WD mice, ns is no differences.

ApoE deficiency (ApoE^−/−^) mice, fed a Western diet, are known to develop atherosclerosis easily [30]. In this study, we provided Western diet-fed ApoE^−/−^ mice with a treadmill, and these mice were trained according to an exercise protocol for 12 weeks (Figure 1A and Supplemental Figure S1A). To confirm that regular exercise training can be protective against atherosclerosis, we first determined the changes in atherosclerosis indicators. As shown in Figure 1B, the body weight of the exercised mice reduced significantly compared with the sedentary groups (*p* = 0.0079). Oil Red O staining of the aortic roots and total aortas of these ApoE^−/−^ mice showed that exercised mice had fewer atherosclerotic lesions and less lipid accumulation (aortic roots, *p* = 0.02; total aortas, *p* = 0.0756) (Figure 1C–E). In addition, serum total cholesterol (TC) and serum triglyceride (TG) levels in the exercised mice decreased (TC, *p* = 0.0433; TG, *p* = 0.0064) (Figure 1F). These data indicated that regular exercise training can inhibit the development of atherosclerosis. As reported, basal BHB levels increased significantly after prolonged exercise [23]. To explore if regular exercise modulated the serum level of BHB in these atherosclerotic mice models, we determined the level of BHB in these mice. We found that after 12 weeks of exercise, the serum level of BHB increased significantly (*p* = 0.0045) (Figure 1G).

### 2.2. Regular Exercise Training Increases the Expression of Lipid Transfer Proteins and Promotes Autophagy in Atherosclerotic Plaques

**Figure 2:**
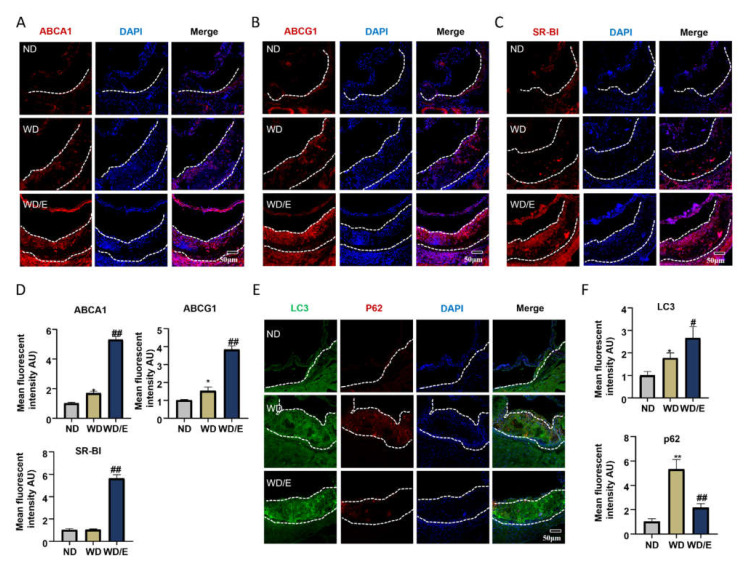
Exercise increases the protein levels of cellular cholesterol transporters and autophagy flux. (**A**–**C**,**E**) Representative immunofluorescence images of aortic root frozen sections from ApoE^−/−^ mice: (**A**) ABCA1 stains (red) and DAPI nuclear stains (blue); scale bars, 50 μm. (**B**) ABCG1 stains (red) and DAPI nuclear stains (blue); scale bars, 50 μm. (**C**) SR-BI stains (red) and DAPI nuclear stains (blue); scale bars, 50 μm. (**E**) LC3 stains (green), p62 stains (red), and DAPI nuclear stains (blue); scale bars, 50 μm. (**D**,**F**) Quantification of immunofluorescence images (n = 6). Data are represented as means ± SEM. * *p* < 0.05, ** *p* < 0.01 vs. ND mice; # *p* < 0.05, ## *p* < 0.01 vs. WD mice.

ABCA1, ABCG1, and SR-BI are all critical cholesterol transporters which are the mechanisms of cholesterol efflux to HDL [12,31]. Enhancing the expression of ABCA1 and other cholesterol transporters is considered antiatherogenic and can inhibit foam cell formation [7]. We hypothesized that exercise might have an effect on the expression of lipid transporters in arteriosclerotic plaques. As shown in Figure 2A–D, we analyzed the protein levels of ABCA1, ABCG1, and SR-BI in the atherosclerotic plaques of mice by immunofluorescence staining, and we found that exercise increased the expression of these lipid transfer proteins (ABCA1, *p* = 0.0059; ABCG1, *p* = 0.0063; SR-BI, *p* = 0.0053). As found previously, cholesterol efflux, mediated by ABCA1, is dependent on autophagy, but it is always inhibited in advanced atherosclerotic lesions [1]. In recent years, previous reports found that regular exercise can mediate the regulation of autophagy in the cardiovascular system [32]. To further investigate the effects of exercise on autophagy in atherosclerotic lesions, we compared the expressions of LC3 and p62, two commonly used autophagy markers, in aortic root plaques. In sedentary mice on a Western diet, p62 intensity was elevated in the lesions, which indicated that autophagy was dysfunctional. After exercise, the expression of LC3 was significantly higher than in the sedentary mice, and the level of p62 decreased dramatically (LC3, *p* = 0.0124; p62, *p* = 0.0054) (Figure 2E,F). In addition, we also investigated the NRF2 expression in the aortic root. We found the expressions of NRF2 were decreased in the exercised mice, which can be regulated by p62 (*p* = 0.0147) (Supplemental Figure S2A). These results suggest that exercise can promote autophagic flux.

### 2.3. BHB Increases the Expression of Lipid Transfer Proteins and Inhibits Macrophage Foam Cell Formation In Vitro

**Figure 3:**
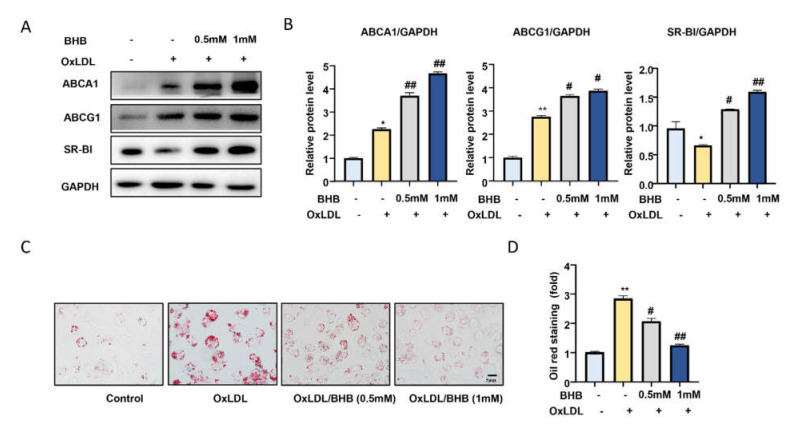
BHB increases the expression of cholesterol transporters and attenuates foam cell for-mation. (**A**) Western blot analysis of the protein expression of cellular cholesterol transporters including ABCA1, ABCG1, and SR-B1 in PMs treated with OxLDL (50 μg/mL) and BHB (0.5 mM, 1 mM) for 24 h. (**B**) Quantification of relative protein expression (n ≥ 3). (**C**) Oil Red O staining of PMs in the presence or absence of OxLDL (50 μg/mL) or BHB for 24 h. Scale bars, 5 μm. (**D**) Quantification of Oil Red O staining (n = 6). Data are represented as means ± SEM. * *p* < 0.05, ** *p* < 0.01 vs. control; # *p* < 0.05, ## *p* < 0.01 vs. OxLDL treated group.

Exercise increased the serum levels of BHB. Previous studies have shown BHB plays an important role in metabolism [25]. To investigate whether an increase in BHB could regulate the expression of these lipid transfer proteins and foam cell formations, we firstly treated peritoneal macrophages (PMs) from ApoE^−/−^ mice with OxLDL in the presence or absence of BHB. As shown in Figure 3A,B, 1 mM BHB markedly upregulated the expression of ABCA1, ABCG1, and SR-BI (ABCA1: *p* = 0.0015; ABCG1: *p* = 0.02; SR-BI: *p* = 0.0006) (Figure 3A,B). We also found that BHB treatment markedly attenuated intracellular lipid accumulation, as analyzed by Oil Red O staining (*p* = 0.0349) (Figure 3C,D) [33]. These results suggest that BHB can enhance the expression of cholesterol transporter proteins in PMs and inhibit foam cell formation.

### 2.4. BHB Ameliorates Autophagy in Macrophages

**Figure 4:**
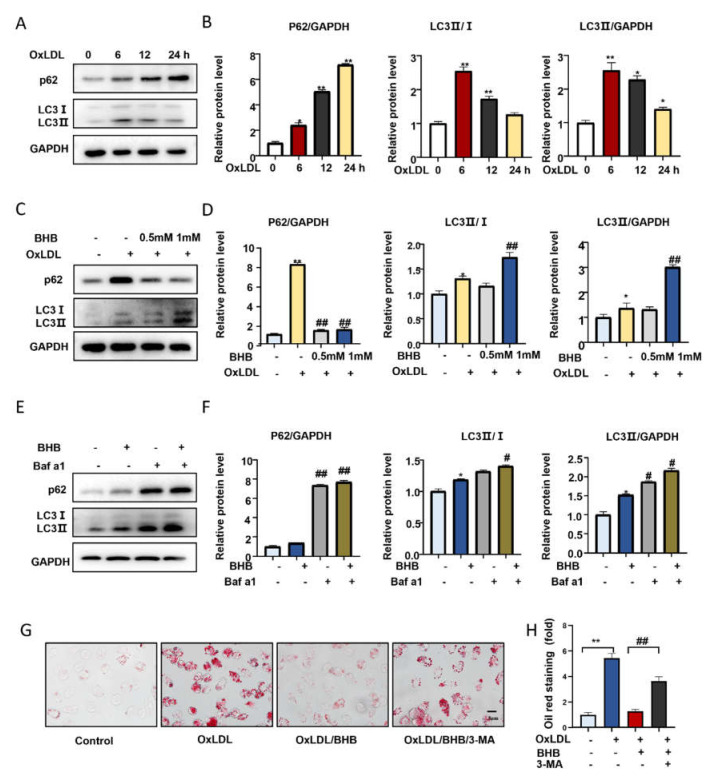
BHB ameliorates autophagy in vitro. (**A**,**C**,**E**) Western blot analysis of p62 and LC3 protein levels in PMs. (**A**) Western blot analysis of the expression of p62 and LC3 in PMs pretreated with or without OxLDL (50 μg/mL) for 6 h, 12 h, and 24 h. (**C**) Western blot analysis of the expression of p62 and LC3 in PMs pretreated with or without OxLDL (50 μg/mL) for 24 h and treated with BHB (0.5 mM, 1 mM) for 24 h. (**E**) Expression of p62 and LC3 in PMs pre-incubated with Baf a1 (200 nM) for 6 h before treatment with BHB (1 mM). (**B**,**D**,**F**) Quantification of relative protein levels (n ≥ 3). (**G**) Oil Red O staining of PMs in the presence or absence of OxLDL (50 μg/mL), BHB (1 mM), or 3-MA (1 mM) for 6 h. Scale bars, 5 μm. (**H**) Quantification of Oil Red O staining (n = 6). * *p* < 0.05, ** *p* < 0.01 vs. control; # *p* < 0.05, ## *p* < 0.01 vs. OxLDL-treated group or BHB-treated group.

We analyzed the protein levels of p62 and LC3 in PMs treated with OxLDL (50 μg/mL) for 6, 12, and 24 h. Western blot results showed that p62 levels time-dependently increased after PMs were incubated with OxLDL (*p* < 0.01 vs. control). The level of LC3II reached a peak at 6 h of OxLDL stimulation, then decreased in a time-dependent manner, suggesting that autophagy was inhibited (*p* = 0.04 vs. control) (Figure 4A,B). BHB (1 mM) treatment for 24 h significantly enhanced autophagy by increasing the expression of LC3II and decreasing the expression of p62 and NRF2 n PMs (p62, *p* = 0.0005; LC3II, *p* = 0.0048) (Figure 4C,D and  Supplemental Figure S2C). To evaluate the effect of BHB on autophagy more accurately, we treated PMs with bafilomycin A1 (Baf a1, 200 nM), which is an autophagy inhibitor at the late stages, for 6 h to inhibit LC3II degradation by lysosomes. We found that LC3II expression was further significantly increased in cells when co-cultured with BHB and Baf a1 (*p* = 0.0034) (Figure 4E,F). Collectively, BHB can enhance autophagosome formation in PMs to promote autophagy flux. In addition, in the presence of the autophagy inhibitor 3-methyladenine (3-MA), which can inhibit autophagosome formation, the effects of BHB on foam cell formation was reversed in PMs, as analyzed by Oil Red O staining (*p* = 0.0086) (Figure 4G,H). These data suggested that BHB could ameliorate autophagy, which is important for reducing lipid accumulation and foam cell formation.

### 2.5. BHB Treatment Is Atheroprotective In Vivo

**Figure 5:**
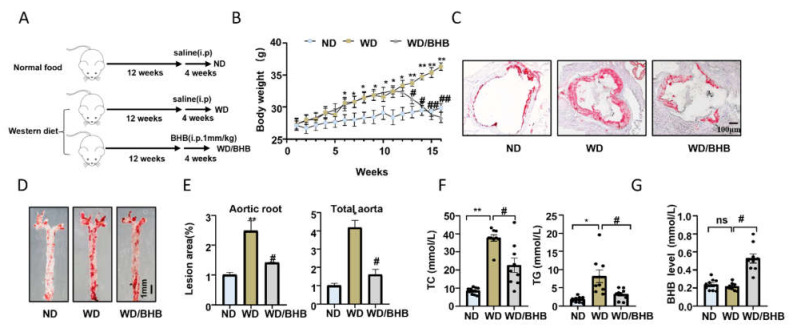
BHB is atheroprotective in ApoE^−/−^ mice fed a Western diet. (**A**) ApoE^−/−^ mice were fed a Western diet for 16 weeks and administered the vehicle or BHB (1 mm/kg daily, i.p.) in the final 4 weeks of the diet. (**B**) Body weight of the mice. (**C**) Representative images of the cross-sections of the aortic root stained with Oil Red O. Scale bar, 100 μm. (**D**) Representative images of the whole aorta stained by Oil Red O. Scale bar, 1 mm. (**E**) Quantification of the cross-sections of the lesion area in the aortic root and whole aorta (n = 6). (**F**) Total cholesterol (TC) and triglycerides (TG) in the serum from the mice (n = 9). (**G**) BHB level in the serum from the mice (n = 9). Data are represented as means ± SEM. * *p* < 0.05, ** *p* < 0.01 vs. ND mice; # *p* < 0.05, ## *p* < 0.01 vs. WD mice, ns is no differences.

To further study the therapeutic effects of BHB on atherosclerosis, 8-week-old ApoE^−/−^ mice were fed a Western diet for 16 weeks to establish an atherosclerosis model, and these mice were intraperitoneally administered BHB in saline at a dose of 1 mmol/kg daily for 4 weeks before being sacrificed (Figure 5A). BHB significantly decreased the bodyweight of these atherosclerotic mice (*p* = 0.0029) (Figure 5B). Oil red O staining showed that BHB reduced the ratio of plaque and lipid accumulation in ApoE^−/−^ mice fed with a Western diet (aortic roots, *p* = 0.0451; total aortas, *p* = 0.0285) (Figure 5C–E). Meanwhile, serum triglyceride and serum cholesterol levels also decreased in the BHB groups (TC, *p* = 0.0093; TG, *p* = 0.0035) (Figure 5F). In addition, we also provided the evidence that the concentration of BHB in the serum increased after a BHB injection in Figure 5G. These results suggest that BHB had an excellent therapeutic effect on ApoE^−/−^ mice.

### 2.6. BHB Increases the Expression of Critical Cholesterol Transporters and Induces Autophagy in Atherosclerotic Plaques

**Figure 6:**
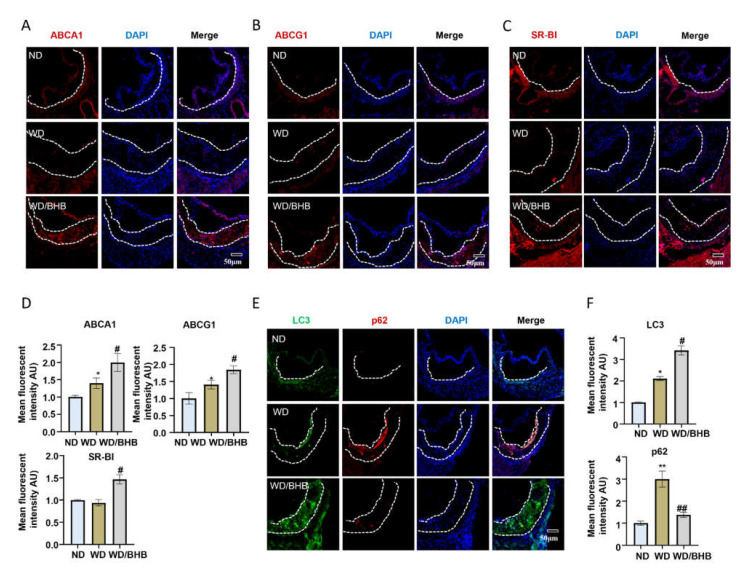
BHB increases the expression of cholestene transporters and induces autophagy. (**A**–**C**,**E**) Representative immunofluorescence images of frozen sections of aortic roots from ApoE^−/−^ mice. (**A**) ABCA1 stains (red) and DAPI nuclear stains (blue). Scale bars, 50 μm. (**B**) ABCG1 stains (red) and DAPI nuclear stains (blue). Scale bars, 50 μm. (**C**) SR-BI stains (red) and DAPI nuclear stains (blue). Scale bars, 50 μm. (**E**) LC3 stains (green), p62 stains (red), and DAPI nuclear stains (blue). Scale bars, 50 μm. (**D**,**F**) Quantification of immunofluorescence images (n = 6). Data are represented as means ± SEM. * *p* < 0.05, ** *p* < 0.01 vs. ND mice; # *p* < 0.05, ## *p* < 0.01 vs. WD mice.

Immunofluorescence staining was used to investigate the effects of BHB on cholesterol transporter proteins and autophagy in atherosclerotic plaques. As shown in Figure 6A–D, the expression levels of ABCA1, ABCG1, and SR-BI were dramatically increased in the aorta roots of BHB-treated ApoE^−/−^ mice (ABCA1, *p* = 0.04326; ABCG1, *p* = 0.0027; SR-BI, *p* = 0.0025). Consistently, the immunofluorescence staining quantification of atherosclerotic lesions in the aortic roots revealed that BHB promotes autophagic flux, where LC3 expression increased and the protein levels of p62 and NRF2 decreased (LC3, *p* = 0.0351; p62, *p* = 0.0008; NRF2, *p* = 0.0015) (Figure 6E,F and Supplemental Figure S2B). Taken together, BHB can promote autophagy and increase the expression of cholesterol transporter proteins in atherosclerotic plaques. The effects of BHB on atherosclerosis in vivo and in vitro are likely to be the mechanisms by which exercise inhibits the development of atherosclerosis.

## 3. Discussion

Our data suggested that exercise inhibited atherosclerosis development by upregulating the level of BHB in the serum of atherosclerotic mice. In vitro and in vivo, we found that BHB can increase the expression of key cellular cholesterol transporters (ABCA1, ABCG1, and SR-BI) and promote autophagy efflux to inhibit the formation of foam cells. This study may provide a new understanding of the anti-atherosclerotic effects of exercise.

We have long known that exercise is beneficial for the prevention and treatment of atherosclerosis. In many clinical studies, exercise can improve lipid metabolism and prevent the oxidation of LDL, which drives the development of atherosclerosis [34,35]. Previous reports also found that exercise can increase eNOS protein expression and decrease superoxide generation to improve NO availability by increasing vascular shear stress, which is important for endothelial function in atherosclerosis [35,36,37,38]. Nascimento et al. reported that exercise training can stabilize atherosclerotic plaques by decreasing the activity of matrix metalloproteinases (MMPs), especially MMP9 [39,40]. In addition, a large amount of evidence has found that regular exercise can reduce other atherosclerosis risks, such as glucose intolerance, hypertension, and insulin resistance [41,42]. However, the precise mechanisms by which exercise can mitigate atherosclerosis are still unknown. In our study, ApoE^−/−^ mice, fed a Western diet, were trained on a treadmill for 12 weeks. Their atherosclerotic plaque area, serum total cholesterol, and triglyceride levels decreased; these results are consistent with previous reports, including some clinical studies [17,43]. However, in the aortic roots of the exercised mice, we found that the expressions of ABCA1, ABCG1, and SRBI increased significantly. These are the key elements of the reverse cholesterol transport pathway. Although some studies in the literature have reported that the expression of ABCA1, and other cholesterol transport proteins, is associated with exercise [44], and that exercise can increase ABCA1 protein levels in the intestine and liver [45], the exact mechanism is unclear. We hypothesized that this effect of exercise has an important relationship with the BHB produced during exercise.

Nowadays, more and more evidence shows that a ketogenic diet and exogenous BHB supplementation can be a promising adjunctive therapy in many diseases, including nonalcoholic fatty liver disease, mood disorders, and children and adolescents with refractory epilepsy [46,47,48]. A ketogenic diet probably creates an unfavorable metabolic environment for cancer cells, which may have potential antitumor effects [49]. In our study, mice maintained high levels of BHB 24 h after the last exercise session, which has non-classical cell signaling functions and can link the external environment with epigenetic gene regulation and cell function [20]. It can bind to the G protein-coupled receptor GPR109A and activate the AMPK signal pathway to exert anti-inflammation effects and prevent the endoplasmic reticulum (ER) stress response and lipid accumulation [25]. BHB treatment can inhibit HDAC3 to promote claudin-5 generation and antagonize diabetes-associated cardiac microvascular hyperpermeability [50]. In addition, BHB can inhibit the activation of NLRP3 inflammasome, which is independent of GPR109A-mediated signaling, HDAC3, autophagy, or other starvation-regulated mechanisms [24]. BHB can penetrate the blood–brain barrier, and it has therapeutic effects on Parkinson’s disease, Alzheimer’s disease, and epilepsy [28,51,52].

However, there are many different opinions on the effects of BHB on atherosclerosis. Castro et al., thought that a ketone body supplement may increase the aortic atherosclerosis burden in mice [53], and, recently, a clinical study conducted by Mu et al. demonstrated that an increase in the circulation level of BHB was associated with increased coronary artery disease severity in a Chinese population. However, there are many experimental and clinical studies providing evidence that ketone bodies, especially BHB, have treatment effects for atherosclerosis. So far, many studies have reported that ketone bodies can reduce insulin resistance and serum resistin levels, which is a proven risk factor for atherosclerosis [54,55]. In addition, because of its molecular mechanisms, some studies have found that BHB can attenuate atherosclerosis via the GPR109A–NLRP3 pathway. It can decrease the proportion of M1-like (classically activated or pro-inflammatory) macrophages in atherosclerotic lesions and inhibit NLRP3 inflammasome activation, which is important for the inflammatory response and cholesterol metabolism via the activation of GPR109A [56,57]. In our study, we also found after exercise and BHB treatment, the recruitment of macrophage and cytokine levels decreased (Supplemental Figure S3A–C). However, the other mechanisms of the therapeutic effect of BHB on arteriosclerosis are not known, or whether it is involved in the anti-atherosclerotic mechanism of exercise. Lipid accumulation and foam cell formation play an important role in the development of atherosclerotic plaques. Lipid-laden macrophages are the main source of foam cells. Therefore, we treated PMs with OxLDL and BHB for 24 h to investigate the effects of BHB on lipid accumulation and foam cell formation in vitro. We found that BHB (1 mM) significantly increased the expression of ABCA1 and ABCG1, and inhibited lipid accumulation and the formation of foam cells, consistent with previous reports. We firstly found that BHB can also promote the protein levels of SR-BI, which is the most important lipid transfer protein in the process of cholesterol efflux.

Before cholesterol efflux from the foam cells, cholesterol must be in its unesterified form. This progress is the rate-limiting step in RCT, and it is independent of autophagy according to recent reports [9,11]. Cholesterol esters that accumulate in cells can be degraded to free fatty acids via the autophagic lysosome pathway and then transported out via ABCA1, etc. [9]. Therefore, autophagy plays a critical role in RCT and the pathogenesis of atherosclerosis. However, autophagy is always disrupted during the progression of atherosclerosis [58]. In aortic root plaques from mice fed a Western diet, we found an accumulation of p62, which is considered to be a marker of autophagy inhibition [59]. In vitro, after OxLDL treatment of PMs for 6 h, LC3II increased, indicating that autophagy was activated to promote the clearance of lipids, but 12 h later, LC3II expression decreased and the protein level of P62 continued to rise, meaning that autophagy was dysfunctional in the PMs. It has been reported that the stimulation of BHB activates the associated autophagic signaling pathway and inhibits vascular calcification via enhanced autophagy [60]. In addition, BHB also can upregulate FOXO1 to activate autophagy in the liver [26]. We found that after exercise, in the aortic root of the ApoE^−/−^ mice, autophagy was promoted when the P62 protein level decreased and the level of LC3 increased. These results may be the effects of BHB. In PMs treated with BHB for 24 h, p62 expression decreased significantly and the protein levels of LC3II increased. Thus, we think that BHB recovered the OxLDL-impaired autophagic flux in PMs. The level of LC3II also increased when PMs were co-cultured with BHB and Baf-a1, so BHB can induce autophagy at the early stage of autophagosome formation. In the presence of the autophagy inhibitor 3-MA, which can inhibit autophagosome formation, the effects of BHB on foam cell formation was reversed in PMs. We also investigated the changes in antioxidant protein NRF2 expression, which is a signal of decreased autophagy and can regulate lipid homeostasis or foam cell formation. As we know, the loss of autophagy caused the accumulation of p62 and caused the robust induction of NRF2 [61,62]. In addition, in the progress of atherosclerosis, NRF2 can promote cholesterol uptake and foam cell formation [63]. We found that BHB treatment could reduce the protein levels of NRF2 in the PMs and atherosclerotic plaques. To sum up, BHB’s ability to ameliorate autophagy is important for inhibiting lipid accumulation and foam cell formation.

To better test the relationship between BHB and exercise in arteriosclerosis, we gave WD mice supplemental BHB and found that they significantly reduced the plaque area, total cholesterol, and triglyceride levels; at the same time, the levels of autophagy and the related lipid transporters were enhanced.

However, we did not take the effects of BHB on other targets, such as endothelial function, into account. We still believe that the increase in BHB levels was the main reason for the effects of exercise on arteriosclerosis. Exercise plays an important role in maintaining cardiovascular system health; for patients with atherosclerosis who have an unstable medical condition, exercise is not a good choice, and our findings may provide a novel treatment of atherosclerosis with BHB, which is the product of exercise and has a strong anti-atherosclerotic effect.

## 4. Materials and Methods

### 4.1. Reagents and Antibodies

The reagents used were as follows: OxLDL (Yiyuan Biotechnologies, Guangzhou, China), BHB (Sigma, Shanghai, China), 3-methyladenine (3-MA), and bafilomycin a1 (Baf a1) (MCE, Monmouth Junction, NJ, USA). The following primary antibodies were used: anti-p62, anti-LC3, anti-ABCG1, and anti-GAPDH antibodies (Proteintech Group, Wuhan, China); anti-NRF2 antibodies (Santa Cruz, Dallas, CA, USA); anti-SR-BI antibodies (Novus Biologicals, Littleton, CO, America); and anti-CD68 and anti-ABCA1 antibodies (Abcam technology, Cambridge, UK). The secondary antibodies used for the Western blot experiments were anti-rabbit and anti-mouse (Vazyme, Nanjing, China). The fluorescent secondary antibodies used for confocal microscopy experiments were as follows: anti-rabbit Alexa Fluor 488-conjugated, anti-mouse Alexa Fluor 488-conjugated, and anti-rabbit Alexa Fluor 594-conjugated, purchased from Thermo Fisher (Thermo Fisher, Waltham, MA, USA).

### 4.2. Animal Studies

The Apolipoprotein E-deficient (ApoE^−/−^) mice were purchased from the Model Animal Research Center of Nanjing University. Mice were housed in an animal facility under controlled conditions (25 ± 2 °C; 12-h light–dark cycle) and were allowed to acquire clean water and food freely. These mice were fed with normal food or a Western diet, which contained 20% fat and 0.15% cholesterol, for 16 weeks. All mice were anesthetized with pentobarbital and then sacrificed at the end of the experiment. Blood samples were collected. Aortas were reserved for atherosclerotic lesion analyses. The use of animal and animal protocols were approved by the Animal Care Committee of Nanjing University (NJU-ACUC) in accordance with the guidelines of the European Directive 2010/63/EU.

### 4.3. Exercise Method and BHB Injection

Apolipoprotein E-deficient (ApoE^−/−^) mice (n = 33) were divided into three groups randomly: the normal diet group (ND), the Western diet group (WD), and the exercised Western diet group (WD/E). The mice assigned to the regular exercise group were trained on a motorized rodent treadmill for 5 days weekly after 4 weeks of the Western diet. The training began at a speed of 10 m/min for 30 min on Day 1, and ended with a speed of 14 m/min for 50 min on Day 5, during the first week, to help mice become familiar with running. These exercised mice were trained for 60 min at a speed of 15 m/min with a break every 15 min during the next 11 weeks [64]. Other ApoE^−/−^ mice (n = 27) were also randomly assigned to the ND and the WD and BHB intervention (WD/BHB). The mice in the BHB intervention group were intraperitoneally injected with BHB (1 mmol/kg, daily) for 4 weeks before being sacrificed. Other groups were injected with saline as a control.

### 4.4. Atherosclerotic Lesion Analysis

At the end of the experiments, the ApoE^−/−^ mice were sacrificed to collect the whole aortas. The aortas were dissected and opened longitudinally under a stereoscope. After the hearts had been surgically harvested, they were embedded in an optimal cutting temperature compound (OCT) and sectioned (10 μm thickness). Whole aortas and cryosections of aortic roots, starting from the proximal aorta to the appearance of the aortic sinus, were stained with Oil Red O for 30 min to evaluate the atherosclerotic lesion areas, while hematoxylin was also used to stain the cell nuclei of the aortic roots [65]. Images of the whole aorta were captured with a SONY camera, and the cryosections were captured under an Olympus light microscope. Image J software was used to quantify the area of the atherosclerotic lesions.

### 4.5. Serum Lipid Measurement

Blood was collected from mice and centrifuged at 5000× *g* for 15 min at 4 °C to acquire the serum. The mouse serum levels of total cholesterol and triglyceride were determined by total cholesterol (TC) and triglyceride (TG) assay kits (Nanjing Jiancheng Bioengineering Institute, Nanjing, China) according to the respective manufacturer’s protocols.

### 4.6. Serum BHB Level Measurement

BHB levels in the serum of the normal food group, the sedentary Western diet, and the exercised Western diet group were measured by using colorimetric assay kits (Cayman Chemicals, Ann Arbor, MI, USA) according to the manufacturer’s protocol [66].

### 4.7. Serum Cytokine Analysis by Enzyme-Linked Immunoassay

Mouse serum cytokine IL-1β and TNF-α levels were determined by the Mouse IL-1β ELISA Kit and the Mouse TNF-a ELISA Kit (MultiSciences, Hangzhou, China) according to the respective manufacturer’s protocols.

### 4.8. Cell Culture

After the administration of the starch broth (6%, i.p.) for 3 days, peritoneal macrophages (PMs) were harvested from ApoE^−/−^ mice by 20 mL ice-cold PBS (3% FBS) lavage of the peritoneal cavities, twice. PMs were centrifuged at 1000 rpm for 5 min at 25 °C and resuspended in RPMI 1640 containing 20% FBS. These cells were plated in 24-well plates and cultured at 37 °C in 5% CO_2_ overnight. Non-adherent cells were removed though washing with PBS, leaving adhered peritoneal macrophages [67]. All PMs were maintained in the culture for 24 hours in RPMI 1640 containing 10% FBS prior to the treatments.

### 4.9. Foam Cell Formation Assays

PMs were seeded in 24-well plates at a cell density of 3 × 10^5^ cells/well and then treated with OxLDL (50 μg/mL) for 24 h to promote the formation of foam cells. BHB (0.5 mM and 1 mM), 3-MA, and Baf A1 were also used to treat the cells for 24 or 6 h. The cells were stained with Oil Red O at 37 °C for 30 min and then visualized under an Olympus light microscope. Image J software was used to quantify the count of foam cells.

### 4.10. Western Blotting Analysis

A RIPA lysis buffer (Beyotime, Shanghai, China) which contained 1% PMSF and 1% protease inhibitor cocktail (Bimake, Houston, TX, America), was used to extract proteins from PMs. The proteins were separated on 10% SDS-PAGE gel and transferred to PVDF membranes. They were blocked with 5% nonfat milk for 2 h in a shaker at room temperature, then incubated with primary antibodies overnight at 4 °C, and incubated with secondary antibodies the next day [68]. Western blots were visualized using ECL electrochemiluminescence (Vazyme, Nanjing, China) and the quantities of proteins were analyzed by Image J software.

### 4.11. Immunofluorescence Staining

Aortic root sections were fixed with 4% paraformaldehyde for 15 min and then permeabilized with 0.2% Triton-X 100 for 10 min. These sections were blocked with 5% bovine serum albumin (BSA) and then incubated with a primary antibody at predetermined concentrations overnight at 4 °C [69]. After incubation with Alexa Fluor 594,488-conjugated secondary antibodies and DAPI for 2 h at room temperature, the samples were mounted with 95% glycerin and observed with a Laser confocal microscope (OlympusFW3000). The staining area and positive protein spots were quantified by Image J software.

### 4.12. Statistical Analysis

Data are presented as means ± SEM (standard error of mean) and represent at least three independent experiments. SPSS 13.0 statistical software (International Business Machines Corporation, Armonk, NY, USA.) and GraphPad Prism 5.0 software were used. Comparisons between two groups used unpaired t-tests, and one-way analysis of variance (ANOVA) tests were performed for comparisons among multiple groups. A p-value of less than 0.05 was considered statistically significant.

## Data Availability

All of the data is available upon request.

## Supplementary Materials

The following are available online at https://www.mdpi.com/article/10.3390/ijms23073788/s1.

## Abbreviations

BHB	β-hydroxybutyric acid
ABCA1	ATP-binding cassette transporter A1
ABCG1	ATP-binding cassette transporter G1
SR-BI	Scavenger receptor class B type I
SRA	Scavenger receptor A
NRF2	Nuclear factor E2-related factor 2
MMPs	Matrix metalloproteinases
NLRP3	NACHT, LRR, and PYD domains containing protein 3
RCT	Reverse cholesterol transport
CE	Cholesteryl ester
ApoE^−/−^	ApoE deficiency
TC	Total cholesterol
TG	Triglyceride
ND	Normal food
WD	Western diet food
PMs	Peritoneal macrophages
3-MA	3-methyladenine
Baf a1	Bafilomycin a1
OxLDL	Oxidative low density lipoprotein
LDL	Low density lipoprotein
AMPK	Adenosine 5‘-monophosphate (AMP)-activated protein kinase
HDC3	Histone deacetylase 3
ER	Endoplasmic reticulum
OCT	Optimal cutting temperature compound
