# Supplementary material for: Exercise Ameliorates Atherosclerosis via Up-Regulating Serum β-Hydroxybutyrate Levels
Source: Int J Mol Sci. 2022 Mar 30;23(7):3788. doi: 10.3390/ijms23073788 (PMC8998237; doi:10.3390/ijms23073788)
Supplement: Supplementary file 1 [file ijms-23-03788-s001.zip › ijms-1623705-supplementary.pdf]

Exercise Ameliorates Atherosclerosis via Up-Regulating Serum β-Hydroxybutyrate Levels

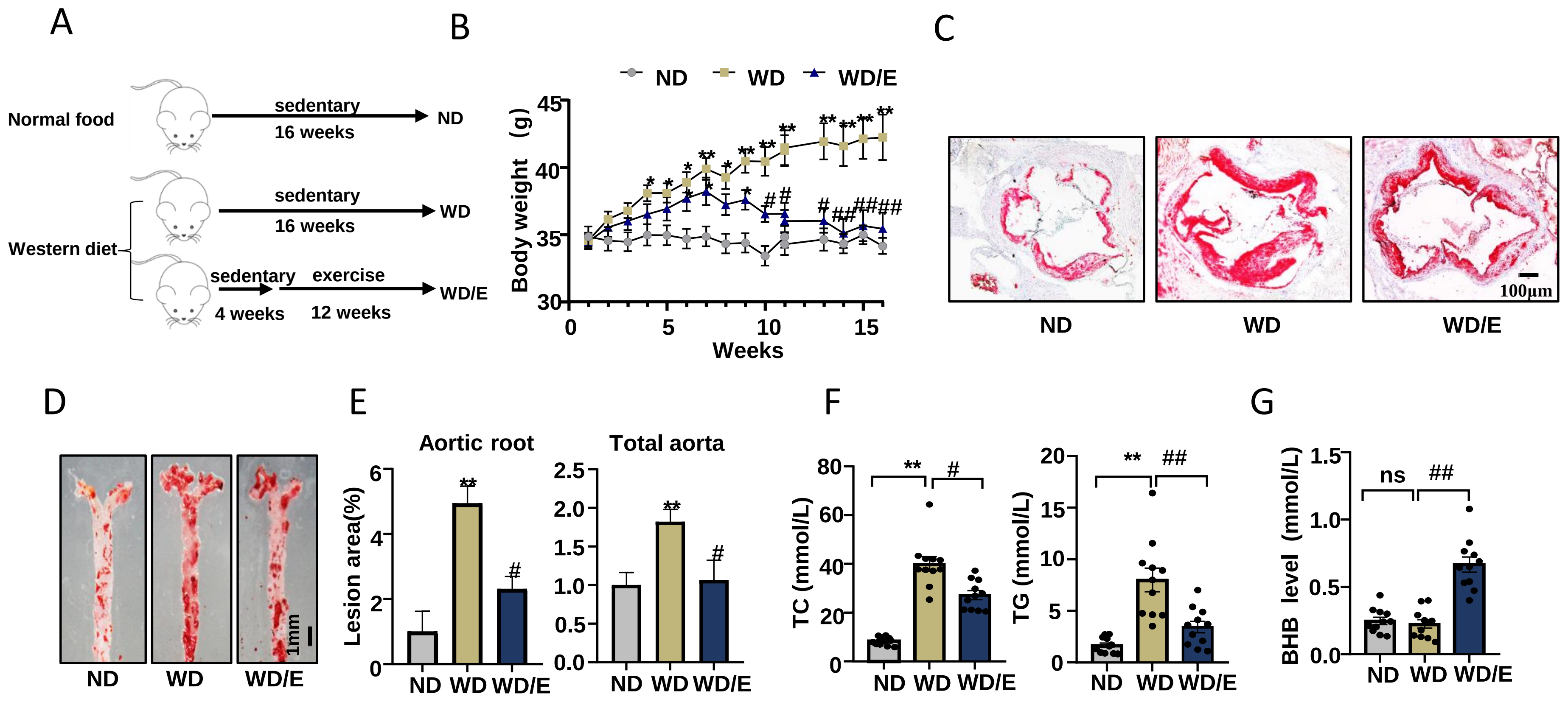

Figure S1. Exercise inhibits the development of atherosclerosis and increases serum BHB levels. A. ApoE-/-mice were fed normal food (ND) or a Western diet (WD), and either had access to exercise (E) for 12 weeks or did not (sedentary) B. Body weight of mice(n=11). C. Representative images of the cross-sections of the aortic root stained with Oil Red O. Scale bars, 100μm. D. Representative images of the whole aorta stained by Oil Red O. Scale bars, 1mm. E. Quantification of cross-sections of the lesion area in the aortic root and whole aorta (n=6). F. Total cholesterol (TC) and triglycerides (TG) in the serum of ApoE-/- mice (n=11). G. BHB levels in the serum from the mice (n=11). Data are represented as means ± SEM. *p < 0.05, **p < 0.01 vs. ND mice, and #p<0.05, ##p<0.01 vs. WD mice, ns is no differences.

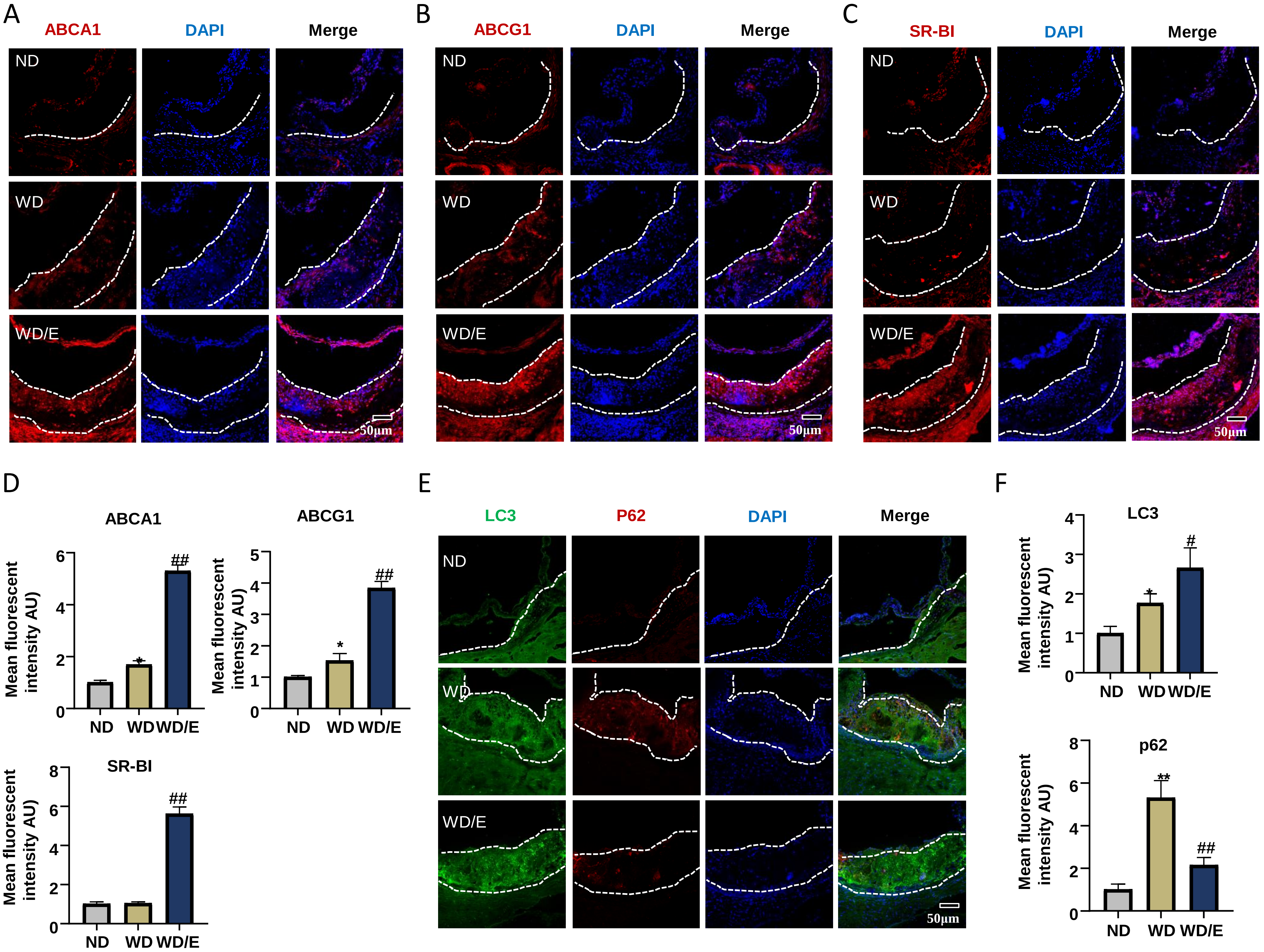

Figure S2. Exercise increases the protein levels of cellular cholesterol transporters and autophagy flux. A, B, C, E Representative immunofluorescence images of aortic root frozen sections from ApoE-/- mice. A. ABCA1 stains (red) and DAPI nuclear stains (blue); Scale bars, 50μm. B. ABCG1 stains (red) and DAPI nuclear stains (blue); Scale bars, 50μm. C. SR-BI stains (red) and DAPI nuclear stains (blue); Scale bars, 50μm. E. LC3 stains (green), p62 stains (red), and DAPI nuclear stains (blue). Scale bars, 50μm. D, F. Quantification of immunofluorescence images (n=6). Data are represented as means ± SEM. *$p < 0.05$, **$p < 0.01$ vs. ND mice; #$p<0.05$, ##$p<0.01$ vs. WD mice.

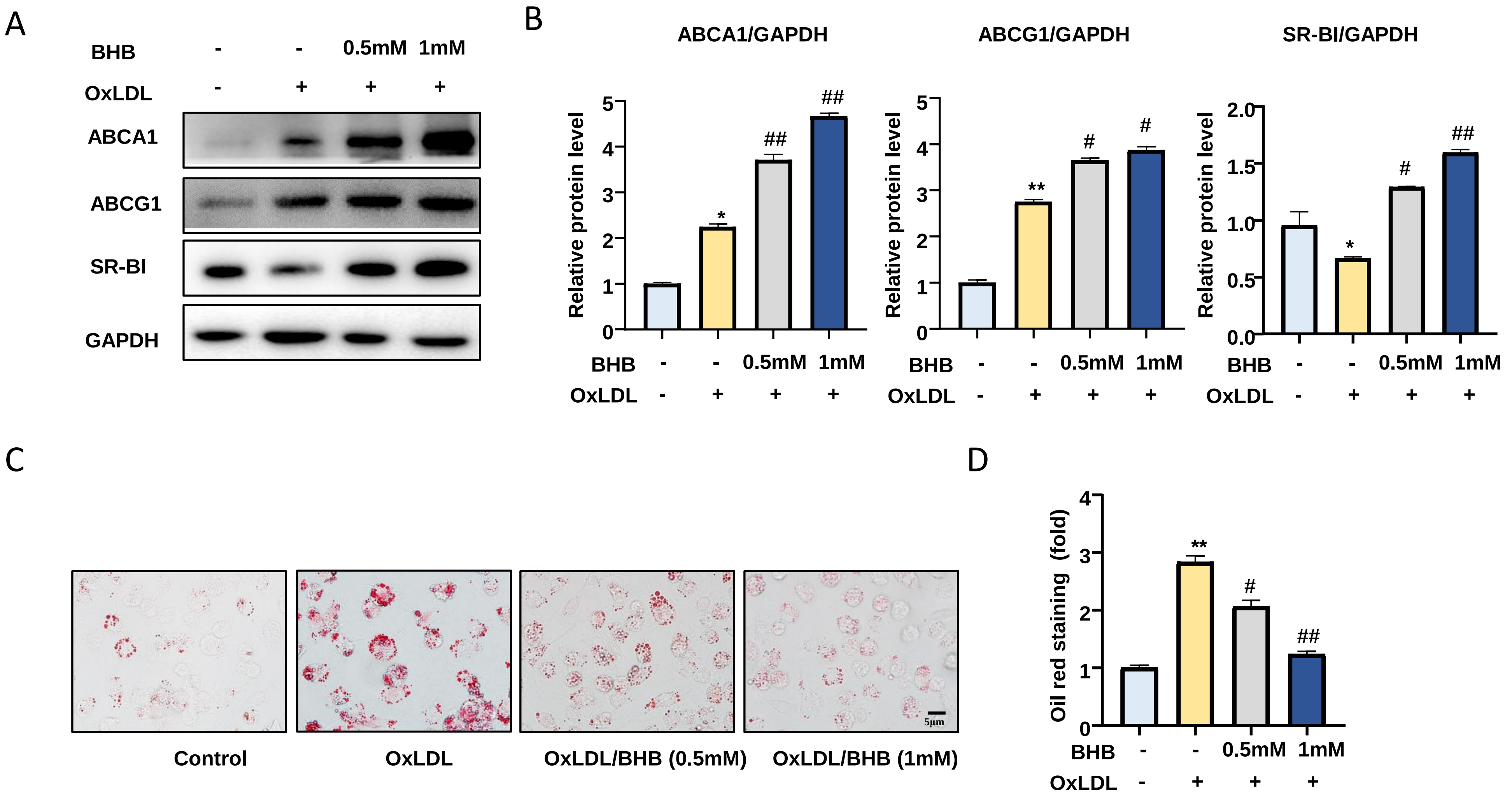

Figure S3. BHB increases the expression of cholesterol transporters and attenuates foam cell formation. A. Western blot analysis of the protein expression of cellular cholesterol transporters including ABCA1, ABCG1 and SR-B1 in PMs treated with OxLDL (50 μg/ml) and BHB (0.5 mM, 1 mM) for 24 hours. B. Quantification of relative protein expression($n \geq 3$). C. Oil Red O staining of PMs in the presence or absence of OxLDL (50 μg/ml) or BHB for 24 h. Scale bars, 5μm. D. Quantification of Oil Red O staining (n=6). Data are represented as means ± SEM. *$p < 0.05$, **$p < 0.01$ vs. control; #$p<0.05$, ##$p<0.01$ vs. OxLDL treated group.

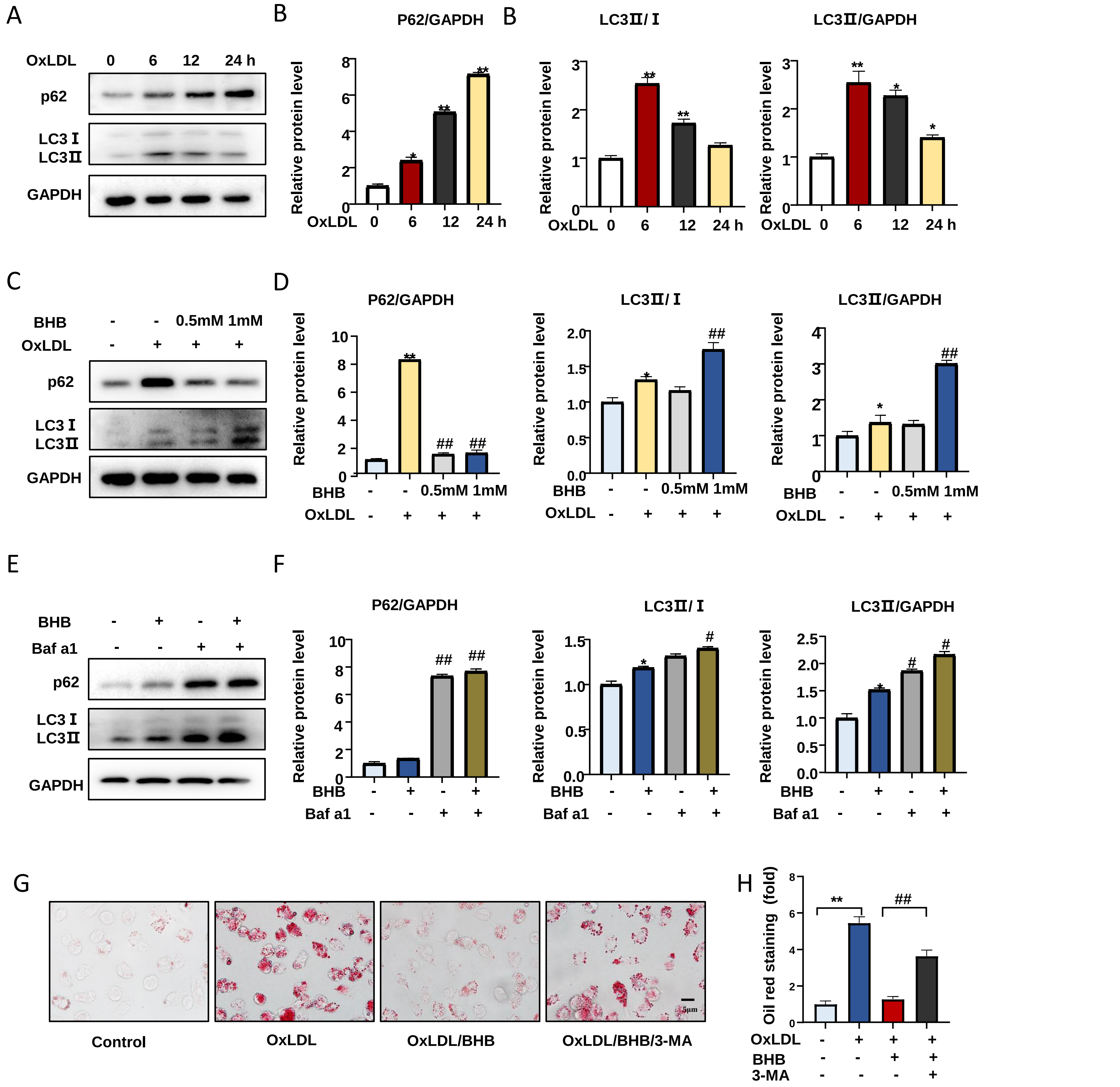

Figure S4. BHB ameliorates autophagy in vitro. A, C, E. Western blot analysis of p62 and LC3 protein levels in PMs. A. Western blot analysis of the expression of p62 and LC3 in PMs pretreated with or without OxLDL (50 g/ml) for 6 h, 12 h, and 24 h. C. Western blot analysis of the expression of p62 and LC3 in PMs pretreated with or without OxLDL (50 μg/ml) for 24 h and treated with BHB (0.5 mM, 1 mM) for 24 h. E. Expression of p62 and LC3 in PMs pre-incubated with Baf a1 (200 nM) for 6 h before treatment with BHB (1 mM). B, D, F. Quantification of relative protein levels(n ≥ 3). G. Oil Red O staining of PMs in the presence or absence of OxLDL (50 μg/ml), BHB (1 mM) or 3-MA (1 mM) for 6 h. Scale bars, 5μm. H. Quantification of Oil Red O staining (n=6). *p < 0.05, **p < 0.01 vs. control; #p<0.05, ##p<0.01 vs. OxLDL-treated group or BHB-treated group.

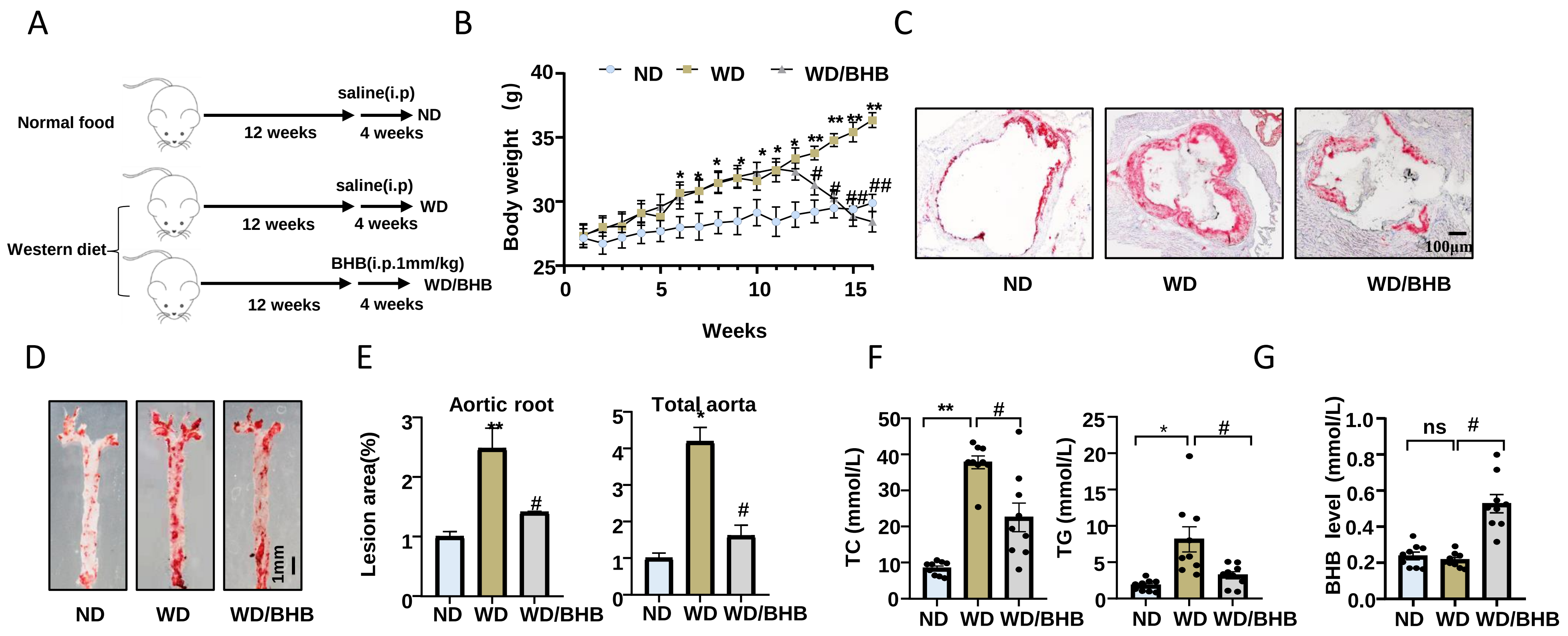

Figure S5. BHB is atheroprotective in ApoE-/- mice fed a Western diet-food. A. ApoE-/-mice were fed a Western diet for 16 weeks and administered the vehicle or BHB (1 mm/kg daily, i.p.) in the final 4 weeks of the diet. B. Body weight of the mice. C. Representative images of the cross-sections of the aortic root stained with Oil Red O. Scale bar,100μm. D. Representative images of the whole aorta stained by Oil Red O. Scale bar, 1mm. E. Quantification of the cross-sections of the lesion area in the aortic root and whole aorta (n=6). F. Total cholesterol (TC) and triglycerides (TG) in the serum from the mice (n=9). G. BHB level in the serum from the mice (n=9). Data are represented as means ± SEM. *p < 0.05, **p < 0.01 vs. ND mice; #p<0.05, ##p<0.01 vs. WD mice, ns is no differences

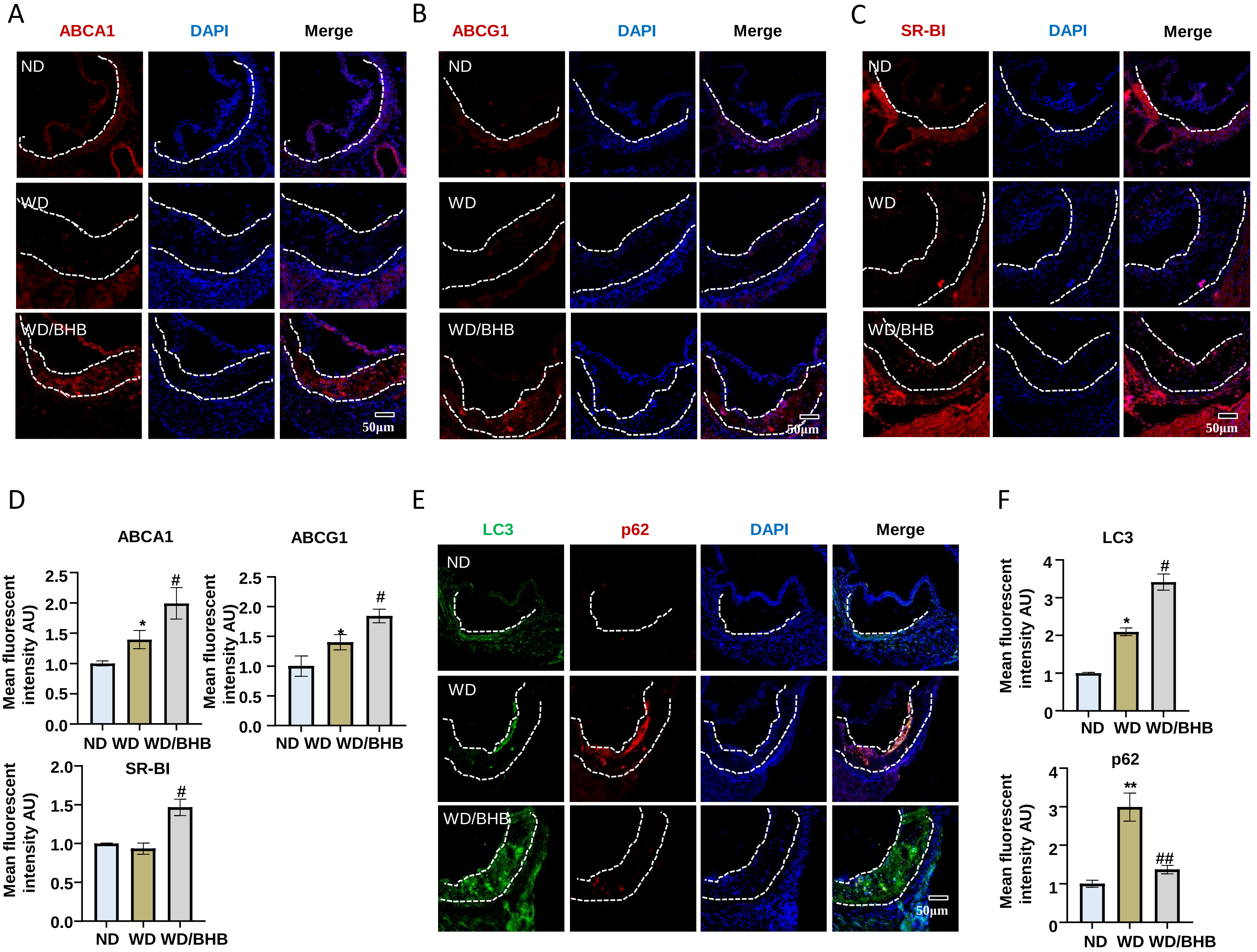

Figure S6. BHB increases the expression of cholestene transporters and induces autophagy. A, B, C, E. Representative immunofluorescence images of frozen sections aortic roots from ApoE-/- mice. A. ABCA1 stains (red) and DAPI nuclear stains (blue). Scale bars, 50μm. B. ABCG1 stains (red) and DAPI nuclear stains (blue). Scale bars, 50μm. C. SR-BI stains (red) and DAPI nuclear stains (blue). Scale bars, 50μm. E. LC3 stains (green), p62 stains (red), and DAPI nuclear stains (blue). Scale bars, 50μm. D, F. Quantification of immunofluorescence images (n=6). Data are represented as means ± SEM. *p < 0.05, **p < 0.01 vs. ND mice; #p<0.05, ##p<0.01 vs. WD mice.
